# Hemoptysis in the Immunocompromised Patient: Do Not Forget Strongyloidiasis

**DOI:** 10.3390/tropicalmed4010035

**Published:** 2019-02-12

**Authors:** Prakash Shrestha, Sean E. O’Neil, Barbara S. Taylor, Olaoluwa Bode-Omoleye, Gregory M. Anstead

**Affiliations:** 1 Covenant Medical Group, Infectious Diseases, Division of Internal Medicine, Lubbock, TX 79410, USA; drprakashshrestha@gmail.com; 2 Texas Center for Infectious Diseases, San Antonio, TX 78223, USA; Sean.oneil@dshs.texas.gov; 3 Department of Medicine, Division of Infectious Diseases, University of Texas Health, San Antonio, TX 78229, USA; TaylorB4@uthscsa.edu; 4 Department of Pathology, University of Texas Health, San Antonio, TX 78229, USA; BodeOmoleye@uthscsa.edu; 5 Medicine Service, Division of Infectious Diseases, South Texas Veterans Healthcare System, San Antonio, TX 78229, USA

**Keywords:** strongyloidiasis, *Strongyloides stercoralis*, hemoptysis, eosinophilia, ivermectin, albendazole, corticosteroids

## Abstract

Strongyloidiasis, due to infection with the nematode *Strongyloides stercoralis*, affects millions of people in the tropics and subtropics. *Strongyloides* has a unique auto-infective lifecycle such that it can persist in the human host for decades. In immunosuppressed patients, especially those on corticosteroids, potentially fatal disseminated strongyloidiasis can occur, often with concurrent secondary infections. Herein, we present two immunocompromised patients with severe strongyloidiasis who presented with pneumonia, hemoptysis, and sepsis. Both patients were immigrants from developing countries and had received prolonged courses of corticosteroids prior to admission. Patient 1 also presented with a diffuse abdominal rash; a skin biopsy showed multiple intradermal *Strongyloides* larvae. Patient 1 had concurrent pneumonic nocardiosis and bacteremia with *Klebsiella pneumoniae* and *Enterococcus faecalis.* Patient 2 had concurrent *Aspergillus* and *Candida* pneumonia and developed an *Aerococcus* meningitis. Both patients had negative serologic tests for *Strongyloides*; patient 2 manifested intermittent eosinophilia. In both patients, the diagnosis was afforded by bronchoscopy with lavage. The patients were successfully treated with broad-spectrum antibiotics and ivermectin. Patient 1 also received albendazole. Strongyloidiasis should be considered in the differential diagnosis of hemoptysis in immunocompromised patients with possible prior exposure to *S. stercoralis*.

## 1. Introduction

An estimated 370 million people in tropical and subtropical regions of the world are infected with the nematode *Strongyloides stercoralis* [1,2,3,4]. (For a map of endemic areas, see Siddiqui et al., 2010 [4]). In the United States, the highest rates of infection occur in immigrants, refugees, travelers, and military personnel who have been to endemic areas and in residents of the Southeastern USA [5,6,7,8].

*Strongyloides stercoralis* differs from other common nematodes by its unique auto-infective lifecycle [9]. Human infection initially results from contact with soil contaminated with human feces containing the infective filariform larvae. The filariform larvae penetrate the skin or mucous membranes and migrate through the veins or lymphatics to the lungs. From there, the larvae migrate through capillaries into the alveoli, move up the trachea, are swallowed by the host, and then localize to the small intestine. There, the female worms mature and lay eggs, which hatch into rhabditiform larvae. Only female adult worms are present in chronic strongyloidiasis; subsequent reproduction occurs by parthenogenesis (the development of an embryo from an unfertilized ovum). Some of the larvae are passed in the stool and begin the external life cycle, while others develop into infective filariform larvae within the host and penetrate the intestinal mucosa and the skin, bypassing the respiratory tract, and establish themselves in the small intestine. By this autoinfection cycle, *Strongyloides* can multiply indefinitely within its host [3,10], and cases of strongyloidiasis have been noted as long as 75 years after leaving an endemic area [11].

## 2. Case Presentations

### 2.1. Case 1

A 46-year old Asian male presented to the emergency department with recurrent hemoptysis. The patient had been diagnosed with dermatomyositis and IgM nephropathy 10 months prior to presentation, and was started on prednisone (50 mg/day; 0.9 mg/kg/day). In an attempt to limit corticosteroid exposure, two weeks after starting prednisone the patient was given azathioprine for two weeks, but he could not tolerate its adverse effects. As a result, he resumed high-dose prednisone (40 mg/day) up to the time of the current hospital admission.

The patient had presented a month prior to the current admission with a cough productive of clear sputum with streaks of bright red blood. A CT scan of the chest at that time showed interstitial thickening and a left lower lobe pulmonary nodule versus atelectasis. Bronchoscopy revealed no endobronchial lesions. Bronchoalveolar lavage fluid grew *Candida albicans* and usual respiratory flora. Serologic testing for infection with *Coccidioides*, *Histoplasma*, *Strongyloides* (IgG by ELISA, ARUP Laboratories, Salt Lake City, UT, USA), and *Cryptococcus* was all negative. An interferon-gamma release assay for the diagnosis of latent tuberculosis conducted one month prior to the current admission was indeterminate, and three sputa for acid-fast bacilli were negative by smear and culture. A urine culture grew *Klebsiella pneumoniae* and *Escherichia coli*. The hemoptysis resolved and the patient was discharged on ciprofloxacin for the urinary tract infection. The patient then presented with hemoptysis of three days duration, associated with fever and chills. He also noticed a rash on his abdomen two days prior to presentation.

The patient was born in Laos and had spent three years in a refugee camp in Thailand before emigrating to the United States 25 years ago. He had lived primarily in San Antonio, Texas, but had travelled to New York City multiple times to work at a landfill. The patient had a 25 pack-year history of smoking, but no history of incarceration or alcohol or recreational drug use.

**Figure 1:**
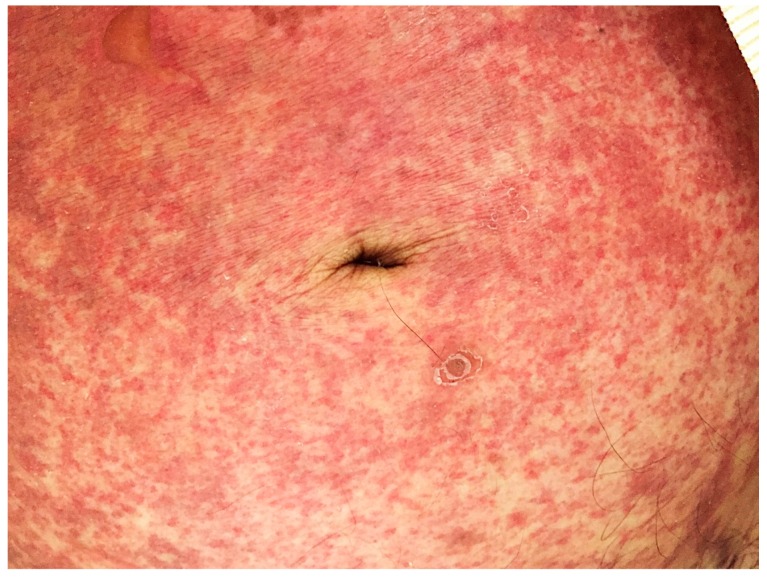
Case 1: Photograph of the peri-umbilical petechial abdominal rash.

On presentation, the patient was lethargic and appeared unwell. Vital signs were: Temperature 38.4 °C, blood pressure 70/40 mm Hg, pulse 125/min, and respiratory rate 20 breaths per minute. On exam, the patient had bilateral coarse crackles, diffuse abdominal tenderness, and a purpuric rash on the anterior trunk extending to the flanks, suprapubic area, groin, and upper thighs (Figure 1). An electrocardiogram showed atrial fibrillation with rapid ventricular response.

**Figure 2:**
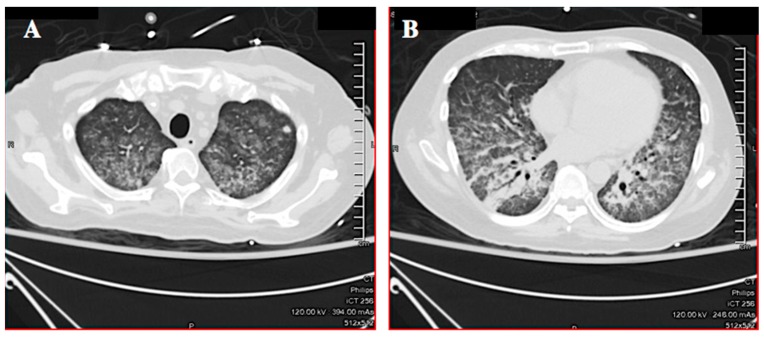
Case 1: CT scan of the chest showing diffuse ground glass airspace opacities, interlobular septal thickening (**A**), and a right middle lobe cavitary lesion (**B**). The airspace opacities suggested diffuse alveolar hemorrhage.

Initial laboratory results were: White cell count 19.5 K/µL (reference range (RR) 3.4–10.4 K/µL) with 51% bands, 38% neutrophils, 3% lymphocytes, 1% eosinophils; hemoglobin 11.8 g/dL (RR 12.8-17.1); platelets 214 K/µL (RR 140-377 K/µL); creatinine 1.2 mg/dL (RR 0.6–1.3 mg/dL), bilirubin 1.2 mg/dL (RR 0.2–1.2 mg/dL); alanine aminotransferase 71 IU/L (RR <46 IU/L); aspartate aminotransferase 47 IU/L (RR <36 IU/L) and alkaline phosphatase 124 IU/L (RR 45–117 IU/L). A CT scan of the chest showed interval development of diffuse ground glass opacities (likely alveolar hemorrhage), interlobular septal thickening, and a single 9 mm right middle lobe cavitary lesion (Figure 2A,B). The patient was admitted to the medical intensive care unit with septic shock. He was started on cefepime, vancomycin, and metronidazole. The next day he required intubation for hypoxemic respiratory failure. Bronchoscopy showed a normal airway with fresh and old blood present, but without an obvious source of bleeding. The differential diagnosis for the hemoptysis considered at the time was tuberculosis, atypical mycobacterial infection, bacterial pneumonia, vasculitis, and *Pneumocystis jiroveci* pneumonia. A punch biopsy of the abdominal rash was performed.

**Figure 3:**
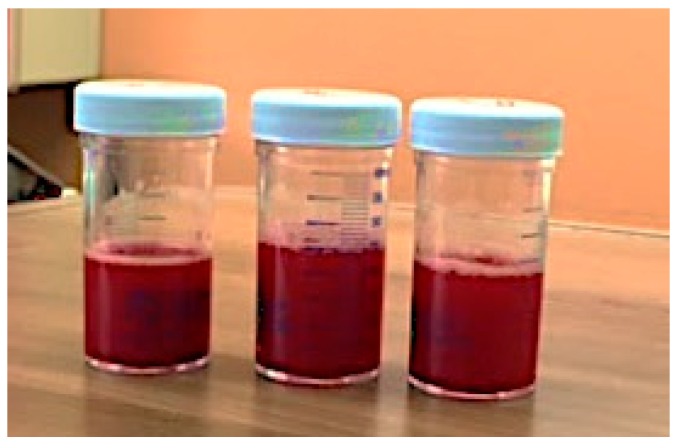
Case 1: Grossly bloody bronchoalveolar lavage fluid.

**Figure 4:**
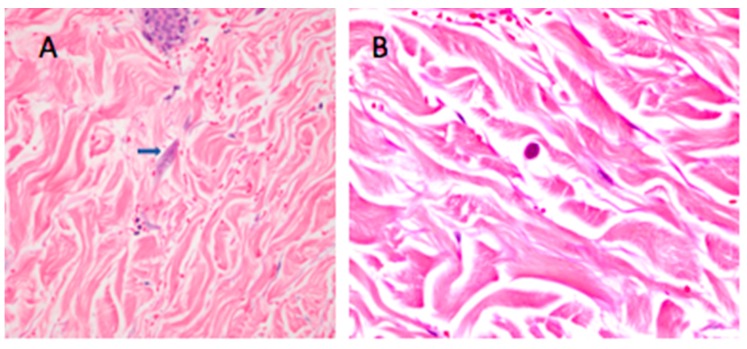
Case 1. (**A**) Longitudinal section of infective filariform *Strongyloides stercoralis* within subcutaneous tissue in skin biopsy of abdominal wall, stained with H&E. Image taken at 200× magnification. Note the absence of inflammatory cells [12]. (**B**) Cross section of infective filariform *Strongyloides stercoralis* within subcutaneous tissue in skin biopsy of abdominal wall, stained with H&E. Image taken at 400× magnification.

Blood cultures from the day of admission grew *K. pneumoniae* and *Enterococcus faecalis.* The grossly bloody bronchoalveolar lavage fluid (Figure 3) grew *Nocardia asteroides* and also revealed the presence of *S. stercoralis* larvae. Histopathologic exam of the skin biopsy showed multiple intradermal helminths consistent with *Strongyloides* (Figure 4). A stool exam conducted on hospital day 13 was also positive for *Strongyloides*.

Starting on hospital day 3, the patient was treated with ivermectin 200 µg/kg/day and albendazole 400 mg twice daily through a nasogastric tube. The patient received albendazole for 21 days and ivermectin for 32 days. The ivermectin was continued until serial sputum and stool studies were negative for the presence of *Strongyloides*. The patient also received cefepime and vancomycin for the polymicrobial bacteremia and trimethoprim-sulfamethoxazole for the nocardiosis. The prednisone dose was decreased to 20 mg per day during the hospitalization. Due to altered mental status, the patient was evaluated by an MRI of the brain and a lumbar puncture, but there was no evidence of CNS infection. The patient was extubated after 10 days of mechanical ventilation. The patient gradually improved and was discharged to a rehabilitation facility in stable condition.

### 2.2. Case 2

The patient is a 36-year old Hispanic man with a history of acute lymphoblastic leukemia that had been diagnosed 14 months prior to the current admission. At that time, he had received induction chemotherapy with cyclophosphamide, vincristine, doxorubicin, dexamethasone, and rituximab (hyper-CVAD-R) and intrathecal chemotherapy, which he finished four months prior to the current admission. He was maintained on monthly 6-mercaptopurine, vincristine, methotrexate, and prednisone (200 mg per day for five days of each month). He had been admitted to the hospital three weeks prior to the current admission for chest pain, malaise, weight loss, and a persistent cough productive of yellow sputum. At that time, he was febrile to 38.4 °C and was initially given vancomycin, piperacillin-tazobactam, and azithromycin. He was found to have diffuse infiltrates on chest X-ray. Sputum culture grew *Pseudomonas aeruginosa* and the patient was transitioned to ciprofloxacin. A nasopharyngeal respiratory pathogen polymerase chain reaction panel (Biofire, Salt Lake City, UT, USA) was positive for *Rhinovirus* and *Enterovirus*. Serologic studies for *Histoplasma*, *Cryptococcus*, *Strongyloides* (IgG by ELISA, ARUP Laboratories) and *Coccidioides* were negative, as were stains of the sputum for fungal and acid-fast organisms. Given the patient’s immunocompromised condition, the diffuse pulmonary infiltrates raised concern for *Pneumocystis* infection. Trimethoprim-sulfamethoxazole (TMP-SMX) and corticosteroids were started empirically with rapid improvement, and the patient was discharged to finish 21 days of TMP-SMX and 14 days of tapering prednisone. The patient presented for the current admission with worsening dyspnea, malaise, fever, and hemoptysis four days after completing ciprofloxacin and TMP-SMX.

The patient was born in Honduras and had emigrated to the United States 16 years prior. The patient lived in San Antonio, Texas, and worked as an electrical technician. He had no animal exposure and no history of incarceration, homelessness, or recreational drug or alcohol use.

On exam, the patient was tachypneic; vital signs were: Temperature 37 °C, pulse 112/min, respiratory rate 30 breaths/min, oxygen saturation of 88% on room air, and a blood pressure 80s/30s mm Hg. Pulmonary exam revealed diffuse rales and expiratory wheezes. The remainder of the exam was unremarkable.

**Figure 5:**
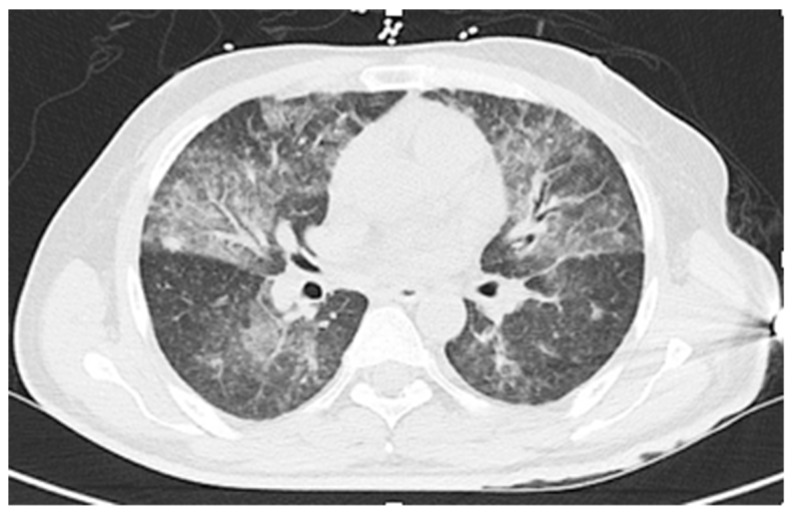
Case 2: CT of the chest showing extensive ground glass and patchy parenchymal opacities throughout the bilateral lungs; the differential diagnosis included opportunistic infections (pneumocytosis, cytomegalovirus), alveolar hemorrhage, and pulmonary edema.

Hematologic results were: White cell count 5.3 K/µL with 36% neutrophils, 6% lymphocytes, 18% eosinophils, 20% bands, and 8% metamyelocytes; hemoglobin 9.7 g/dL; and platelets 138 K/µL. Serum chemistry values were: Sodium 120 mmol/L (RR 135-145 mmol/L) and bilirubin 1.6 mg/dL (0.2–1.2 mg/dL); creatinine, alanine aminotransferase, aspartate aminotransferase, and alkaline phosphatase levels were all within normal limits. A CT scan of the chest showed interval worsening as compared to three weeks prior, with extensive ground glass and patchy parenchymal opacities throughout the bilateral lungs, suggestive of multi-lobar *Pneumocystis* pneumonia (see Figure 5).

The patient was admitted to the intensive care unit with septic shock. The initial differential diagnosis for the patient’s respiratory distress included viral or bacterial pneumonia, vasculitis, malignancy, and *P. jirovecii* pneumonia. He was started on cefepime, vancomycin, TMP-SMX, metronidazole, and azithromycin, and received five liters of normal saline and norepinephrine for blood pressure support. Prednisone was held. Sputum cultures again grew *P*. *aeruginosa* with the same susceptibility pattern as in previous cultures. A nasopharyngeal swab for viral respiratory pathogens was again positive for *Rhinovirus* and *Enterovirus*. Sputum cytology was also obtained to evaluate for malignancy. The patient improved after 24 days and was transferred to the ward.

**Figure 6:**
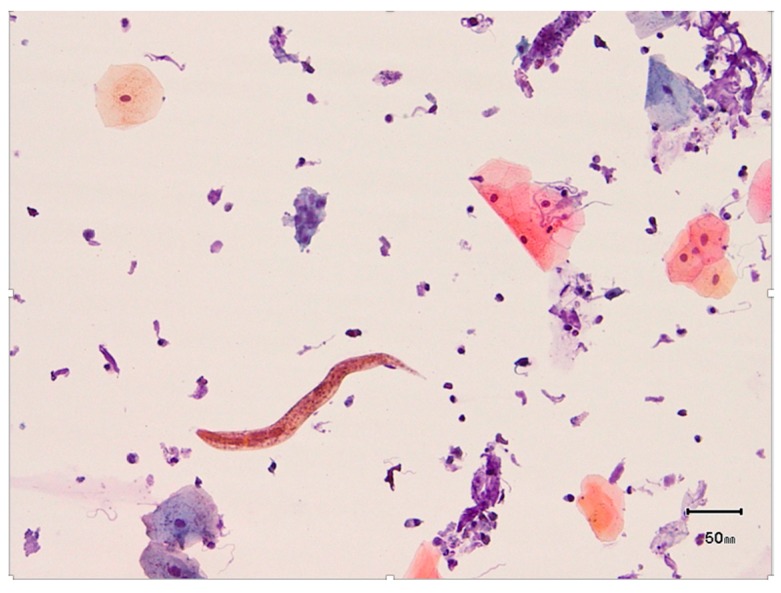
Case 2: Cytologic exam of sputum showing *S. stercoralis*.

Sputum cytology revealed helminth larvae consistent with *S. stercoralis* (Figure 6). The patient was started on ivermectin (200 µg/kg/d) and continued to improve. Sputum cultures also grew *Aspergillus flavus* and *Candida tropicalis*. Bronchoscopy was performed and the lavage fluid grew *A. terreus*; *C. guilliermondii* grew from tissue from a transbronchial biopsy, and he was started on voriconazole. He was discharged in stable condition. At clinic three weeks later, the patient reported a constant dull headache and a lumbar puncture showed neutrophilic pleocytosis; a CSF culture grew *Aerococcus viridans*. He was successfully treated with a 14-day course of vancomycin. He continued ivermectin until two weeks of serial sputum and stool samples were negative for the presence of *Strongyloides* (64 total days of treatment).

## 3. Discussion

Hemoptysis is the expectoration of blood originating in the lower respiratory tract. Hemoptysis due to alveolar hemorrhage has an extensive differential diagnosis of infectious, autoimmune, neoplastic, cardiovascular, and miscellaneous causes. The most frequent conditions causing hemoptysis are bronchiectasis, tuberculosis, mycoses, necrotizing pneumonia, and malignancy [13]. In the immunocompromised patient with hemoptysis, an infectious cause is a major concern, including infection with cytomegalovirus, adenovirus, *Aspergillus*, *Mycoplasma*, *Legionella*, and *Strongyloides* [14]. For those patients who have lived in a developing country, strongyloidiasis should be included in the differential diagnosis for hemoptysis. Pulmonary strongyloidiasis typically has an asthma-like presentation, but 10% of patients suffer hemoptysis [15]. Diffuse alveolar hemorrhage from strongyloidiasis may have a fatal outcome [16,17,18]. We cannot rule out that the pulmonary co-infections (with *A. terreus*, *A. flavus*, and *N. asteroides*) may have contributed to the hemoptysis; however, compared with *Strongyloides*, reports of these other pathogens causing pulmonary hemorrhage are infrequent.

In immunocompetent persons, *S. stercoralis* may cause mild intestinal discomfort, urticaria, or asymptomatic carriage for decades. However, in patients with iatrogenic or disease-induced immunosuppression, including human T-lymphotrophic virus-1 (HTLV-1) infection, corticosteroid use, organ transplantation, or tumor necrosis factor antagonist use, potentially fatal hyperinfection syndrome or dissemination may occur. In these patients *Strongyloides* infection can cause more severe symptoms, including nausea, diarrhea, gastrointestinal and alveolar bleeding, weight loss, small bowel obstruction, and severe abdominal pain as adult parasites invade the duodenal and jejunal mucosa. *Strongyloides* hyperinfection syndrome is an accelerated autoinfection process, with proliferation of the previously stable population of worms to a level which adversely affects the health of the host. Detection of abundant larvae in the stool or sputum is indicative of hyperinfection [19]. During disseminated strongyloidiasis, large numbers of worms (primarily filariform larvae) reach extra-intestinal organs, including the skin, lungs, peritoneum, liver, kidneys, and central nervous system. A pathognomonic sign of disseminated strongyloidiasis is larva currens, a serpiginous cutaneous lesion of the buttocks, groin, perineum, and/or trunk; however, it is not a common finding [20]. A peri-umbilical purpuric rash, as in Case 1, has been previously described in disseminated strongyloidiasis and is a sign of poor prognosis [12,21]. The purpura has been attributed to the invasion of the dermis by larvae that have migrated through the vessel walls. The periumbilical distribution may be due to retrograde venous migration. Additionally, the larvae may penetrate into the skin from the abdominal cavity, following migration through the wall of the colon [12]. However, there is typically a blending of hyperinfection and dissemination, so it is simpler to categorize strongyloidiasis as “uncomplicated” or “severe" [4,5].

Secondary infections are common in severe strongyloidiasis due to bacterial translocation from the gut by the hematogenously migrating larvae and underlying immunosuppression [3,11,22,23]. The bacterial infections (primarily bacteremia, pneumonia, and meningitis) that often accompany severe strongyloidiasis are a major factor in the shock, multi-organ failure, and death due to this infection [23]. Chest radiographic findings in severe strongyloidiasis are non-specific; there may be pulmonary infiltrates, consolidation, and occasional cavitation or abscess formation. The variable radiographic appearance is due to concurrent superinfection by other organisms [4].

Several immunosuppressive medications and underlying conditions are associated with *Strongyloides* hyperinfection [24]. Due to the risk of hyperinfection and dissemination in the immunocompromised patient, American and British guidelines recommend that all patients from endemic areas be screened serologically for *Strongyloides* infection prior to commencement of immunosuppressive therapy [24,25]. Patients found to have strongyloidiasis upon screening can be treated with ivermectin in order to prevent future hyperinfection after immunosuppression [26]. It has been proposed that the increased risk of hyperinfection in patients taking corticosteroids is not due to immunosuppression per se but that corticosteroids mimic the ecdysteroid molting hormones of *Strongyloides* [27]. Thus, the administration of exogeneous corticosteroids promotes the transformation of the rhabditiform larvae into invasive filariform larvae [3].

A diagnosis of strongyloidiasis is typically confirmed if the rhabditiform larvae are seen with a microscopic exam of stool specimens or respiratory samples. However, a single stool exam is diagnostic in only one-third of patients. Serial stool examinations increase the sensitivity of stool exams but may be impractical. It is estimated that seven stool exams would provide close to 100% sensitivity [4,28]. In a series of patients with known chronic strongyloidiasis (i.e., passing of larvae in the stools), 95% were serologically positive and 83% had eosinophilia [29]. However, in patients with severe strongyloidiasis that have received corticosteroids or other immunosuppressive agents, serologic tests may be falsely negative [30]. During hyperinfection, eosinophilia is usually absent [5,31], but patients that maintain eosinophilia during hyperinfection have a better prognosis [23,32]. The agar plate culture of feces method (with observation of the tracks of bacteria arising from migrating larvae) has the highest detection rate for strongyloidiasis in immunocompromised patients, whereas serologic testing has a low yield in this setting [33]. In addition to the usual methods of sputum and stool detection, skin biopsy or observing the larvae in bronchoalveolar lavage fluid, ascites, pleural fluid, urine, or cerebrospinal fluid may also be diagnostic [4].

Acute uncomplicated strongyloidiasis is treated with one to two doses of ivermectin, with reports of over 90% efficacy [1]. However, recent studies using molecular methods for detection have suggested that traditional dosing of ivermectin may not be sufficient to eradicate *Strongyloides* [34]. An alternative treatment is albendazole 400 mg twice daily for seven days, although the parasitologic cure rate (63.3%) is lower than that of ivermectin [35,36]. However, the optimal treatment for critically ill patients with severe strongyloidiasis is uncertain, and data regarding the ideal drug(s), doses, duration of treatment, and route of administration are limited. In an analysis of 244 cases of severe strongyloidiasis abstracted from the medical literature, it was uniformly fatal without treatment. Ivermectin or thiabendazole (currently unavailable in the USA) administration reduced the mortality rate to 47% and 51%, respectively, but in the albendazole group, 73% died [2,36]. However, not all published case series of severe strongyloidiasis carry a high mortality rate; in one series of nine patients with severe *Strongyloides* infection with respiratory failure, the mortality rate was 33% [32]. In another group of 16 patients with severe strongyloidiasis, in which all the patients were treated with ivermectin, the mortality rate was significantly lower (11.1%) [37].

In severe strongyloidiasis some experts recommend five to seven days of ivermectin or combining ivermectin with albendazole until the patient responds clinically and daily stool examinations have been negative for at least two weeks (one autoinfection cycle), with ongoing monthly ivermectin if the patient remains immunosuppressed [10,24,38]. Rectal ivermectin has been used in patients with severe strongyloidal colitis [39], but rectal administration may not achieve therapeutically sufficient serum levels of the drug [40]. In cases of severe strongyloidiasis in which anti-helminthic drug administration by the enteric route is not feasible due to ileus, subcutaneous injection of the parenteral veterinary formulation of ivermectin has been advocated [36,38]. Whenever possible, immunosuppressive agents should be discontinued or decreased to lowest possible dose. In particular, continued corticosteroid use is associated with a poor outcome. In severe strongyloidiasis, broad spectrum antibiotics that cover enteric gram-negative bacteria should also be administered empirically during the period of severe illness or for the standard duration necessary to treat any diagnosed intercurrent infections. There is no definitive test of cure following treatment of strongyloidiasis, but in those patients with pre-treatment eosinophilia and positive IgG serologic tests, resolution of eosinophilia and a decline in IgG antibody levels after an average of 96 and 270 days, respectively, indicates successful treatment [8,24,29].

## 4. Conclusions

These cases illustrate that patients from *Strongyloides*-endemic areas should be serologically screened prior to commencement of immunosuppressive therapy and receive ivermectin if such screening is positive. Furthermore, strongyloidiasis should be considered in the differential diagnosis for hemoptysis in immunocompromised patients that have lived in or traveled to endemic areas. In immunocompromised patients, eosinophilia and serologic studies are not sensitive diagnostic tests [31], and the examination of stool, sputum, bronchial washings, and cutaneous biopsy specimens may be necessary to afford the diagnosis. The patients of these two cases had classic risk factors for the development of severe *Strongyloides* infection. Both patients were immigrants from endemic countries and both had been treated with extended courses of corticosteroids. The patient of Case 1 received high-dose corticosteroids for nine months for dermatomyositis and IgM nephropathy prior to presentation. The patient of Case 2 received scheduled cytotoxic chemotherapy including corticosteroids for over one year. Both patients had prodromal respiratory illnesses in the month prior to their critical illness, but in each case, the diagnosis of strongyloidiasis was not initially pursued beyond serologic testing. In both cases, serological tests were negative and in Case 1 either the immunocompromised state of the patient or the high doses of prednisone blunted the eosinophilic response often observed in immunocompetent persons with strongyloidiasis. In Case 2, eosinophilia was retrospectively identified by the infectious diseases specialists in the periods between cycles of chemotherapy administration. Both patients suffered multiple concurrent infections (*Klebsiella/Enterococcus* bacteremia and *Nocardia* pneumonia in Case 1 and *Pseudomonas*/viral/fungal pneumonia and *Aerococcus* meningitis in Case 2). Aggressively seeking and treating concurrent infections in patients with strongyloidiasis is necessary to optimize patient outcomes.
